# Recent advances in understanding Type 1 Diabetes

**DOI:** 10.12688/f1000research.7356.1

**Published:** 2016-01-27

**Authors:** Gustaf Christoffersson, Teresa Rodriguez-Calvo, Matthias von Herrath

**Affiliations:** 1 Type 1 Diabetes Center, La Jolla Institute for Allergy and Immunology, La Jolla, California, 92037, USA; 2 Novo Nordisk Diabetes Research & Development Center, Seattle, Washington, 98109, USA

**Keywords:** Type 1 Diabetes, Beta Cells, Environmental Triggers, Beta Cell Mass, MHC-I expression, Insulitis

## Abstract

Type 1 diabetes is a multifactorial disease in which genetic and environmental factors play a key role. The triggering event is still obscure, and so are many of the immune events that follow. In this brief review, we discuss the possible role of potential environmental factors and which triggers are believed to have a role in the disease. In addition, as the disease evolves, beta cells are lost and this occurs in a very heterogeneous fashion. Our knowledge of how beta cell mass declines and our view of the disease’s pathogenesis are also debated. We highlight the major hallmarks of disease, among which are MHC-I (major histocompatibility complex class I) expression and insulitis. The dependence versus independence of antigen for the immune infiltrate is also discussed, as both the influence from bystander T cells and the formation of neo-epitopes through post-translational modifications are thought to influence the course of the disease. As human studies are proliferating, our understanding of the disease’s pathogenesis will increase exponentially. This article aims to shed light on some of the burning questions in type 1 diabetes research.

## Introduction

For many years, type 1 diabetes (T1D) was defined as an autoimmune disease in which autoreactive T cells escape negative selection and destroy the insulin-producing beta cells. Nowadays, we know that the disease is complex, involving many immune and non-immune elements, many of which remain obscure. Several potential environmental triggers of the disease have been described, with viral infections at the top of the list. Alas, still no definitive evidence has been found that directly proves them to be the causative agents of T1D.

Recent access to pancreatic tissue from T1D subjects (thanks to the efforts of several biobanks around the world) has shown great variability in beta cell mass decline in patients on their first year post-diagnosis, arguing for the existence of an unknown amount of remaining beta cells in the pancreas. Moreover, insulitis, one of the hallmarks of T1D, has been shown to be a heterogeneous lesion in humans, suggesting that the immune attack is not orchestrated entirely by T cells, as the pace of destruction is different for every individual. The innate immune system and non-specific inflammation have been increasingly linked to T1D and are contributing factors to the pathogenesis of this complex disease. Thus, T1D might not be a fully T cell-mediated disease, as it seems not to be entirely antigen driven. Our knowledge of the abundance and role of antigen-specific T cells is very limited. T cells autoreactive against known beta cell antigens are very scarce in both peripheral blood and the pancreas of patients with T1D. This suggests that there are other non-antigen-specific cells involved or that we do not know the full specificities of the immune infiltrate. In this regard, efforts are being made by the scientific community to discover new autoantigens in T1D, among which are possible post-translational modifications (PTMs) that lead to the formation of new antigens not previously recognized by the immune system. In this brief review, we navigate through the pathogenesis of T1D, from possible triggers to classic models and dogmas of the disease, summarizing recent findings in prioritized areas of the field.

## Viral infection and other possible triggers: how do we deal with the environment?

T1D is an inherently difficult disease to study. The main destructive autoimmune events in the pancreas and the trigger of these may pre-date diagnosis by several years. This discrepancy in time between disease triggering and diagnosis makes it very difficult to draw conclusions about the cause of the disease from samples taken from patients with T1D; it’s like painting a portrait of a person you barely see the shadow of.

Over the years, epidemiological studies have shown a significant correlation between the environment and the development of T1D: there is currently a 3% annual increase in incidence, which cannot be explained by genetic predisposition (e.g. MHC [major histocompatibility complex] class II haplotypes) ^1, 2^, there is high heterogeneity in the geographical distribution of the disease ^3^, and, in the offspring of immigrants, T1D incidence rate will be approximately that of their new country of residence ^4^.

A wide range of environmental factors have been shown to correlate with T1D development in susceptible individuals, such as dietary factors (e.g. cow’s milk and gluten ^5, 6^), toxins (e.g. N-nitroso compounds ^7^), stress ^8^, and enteroviral infection (e.g. coxsackievirus B ^9^). The composition of the gut microbiome is emerging as another major player in autoimmunity. Several animal studies indicate that alterations in the intestinal microbiota are associated with the development of autoimmune diabetes. In bio-breeding rats, a difference in microbiota composition was seen prior to onset of T1D in animals that are prone to the disease versus ones that are protected ^10^, and a transferable protection has also been observed in non-obese diabetic mice lacking MyD88 (myeloid differentiation primary response gene 88), an essential signal transducer in Toll-like receptor signaling ^11, 12^. Studies showing an influence of the human microbiome and the development of T1D have been few and limited in numbers of study subjects ^13^. Recently, however, a study using longitudinal data from 33 Finnish and Estonian children reports that a shift in microbiota occurred after seroconversion but before onset of T1D ^14^. Still, a direct relationship between the gut and the development of T1D in humans remains to be established. Nonetheless, these recent findings prompt further investigation into the correlation of the microbiome and the increased gut permeability seen in T1D subjects ^15^ and its prospective contribution to the entry of possible T1D-promoting agents such as enterovirus.

Despite the plethora of studies that have implicated virus as a major environmental factor in the development of T1D, evidence remains indirect. A recent report took a proteomics approach to assess the anti-viral response in 42 young patients with T1D ^16^. Even though the array of viruses was not exhaustive, only one strain, Epstein-Barr virus, correlated with recent-onset T1D. In an effort to attain highly relevant tissue for the main purpose of investigating viral influence in disease onset, pancreas tail resections from patients with recent-onset (3 to 9 weeks after diagnosis) T1D were collected as part of the Diabetes Virus Detection (DiViD) study ^17^. In these samples, evidence for an ongoing enteroviral infection was detected in all six patients through the expression of enteroviral capsid protein 1 (VP1) in some of the remaining islets ^18^. Causality has yet to be proven in the case of viral infection, but recent advances in molecular techniques and access to highly relevant tissues (as illustrated above) might help close the temporal gap between the trigger of the disease and diagnosis.

## What does the type 1 diabetes scenario look like nowadays? The relapsing-remitting model

In 1986, George S. Eisenbarth proposed that T1D was a “genetically programmed autoimmune disease” ^19^. In his view, six different stages could be distinguished: (I) genetic susceptibility, (II) triggering event, (III) active autoimmunity, (IV) immune abnormalities accompanied by loss of glucose-stimulated insulin secretion, (V) overt diabetes with few remaining beta cells, and (VI) complete loss of beta cells. This model was presented in parallel with the stages of beta cell destruction; normal beta cell mass remained in stage I, whereas in stage V, at onset, only 10% of beta cell mass was left. At stage VI, a complete loss was described. Nonetheless, it was one of the first attempts to explain the pathogenesis of T1D and beta cell decline that provided a useful framework to conduct clinical research studies and was widely accepted at the time. The linear beta cell mass decline model was adopted for many years; this was due in part to the lack of proper tools to find a good correlate for remaining beta cell mass and the absence of longitudinal studies capable of following patients during the pre-diabetic phase and after diabetes onset.

There has been some debate as to whether or not the linearity of Eisenbarth’s model was accurate. Other models depicting variable beta cell mass decay have been described ^20^. Accordingly, these models have tried to include several scenarios in which genetic predisposition and environmental triggers might play different roles ^21^. Among these, the relapsing-remitting hypothesis has been well accepted as an alternative scenario in the disease’s pathogenesis ^22^. In this model, a fluctuation in beta cell mass is present over time as a consequence of different waves of beta cell killing in a complex battle among regulatory elements, autoreactive cells, beta cell survival, and their subsequent feedback systems.

After three decades of contemplating the idea of a linear, variable, or relapsing-remitting model, or a combination of these, it has become more evident that these models might be partially right or partially wrong, depending on which stage of the disease we are looking at. A recent study by Herold *et al.* ^23^ showed the presence of elevated levels of unmethylated insulin ( *INS)* DNA and their correlation with beta cell death in a cohort of at-risk patients. Beta cell killing starts long before diabetes onset, but interestingly the increase in beta cell death is subtle and sporadic prior to diagnosis, with only a mild repercussion on insulin secretion. However, a marked increase in unmethylated *INS* DNA is seen closer to the time of diagnosis, declining after the development of hyperglycemia. This would suggest that both the relapsing-remitting and the linear models could be applied to the pre-diabetes and peri-diagnosis periods, respectively, with small cyclic waves of beta cell destruction followed by an abrupt fall of beta cell mass at diagnosis. This is in agreement with previous publications in which beta cell function seems stable for many years, declining only around the time of diagnosis ^24– 26^. In accordance, a recent publication by Fisher *et al.* ^27^ revealed an increase in beta cell death at onset, which diminished at 8 weeks post-diagnosis, a phenomenon that appeared to be specific to the onset of T1D. In chronic infections, the persistent exposure to antigen is associated with an exhaustion of CD8 ^+^ T cells and the failure to clear the virus ^28^. Similarly, CD8 ^+^ T cell exhaustion has also been observed in autoimmunity (including T1D), in part explaining the relapsing-remitting nature of these diseases. After a while, the immune system can revert the exhaustion and again have fully functional autoreactive CD8 ^+^ T cells ^29^ capable of continuing their attack on the beta cells. Ultimately, a critical amount of beta cell mass is lost and the death of beta cells cannot be compensated, leading to the development of hyperglycemic symptoms and the rapid beta cell demise around the time of diagnosis.

Several scenarios of beta cell decline have been described in the past; however, the ideal model would also take into account clinical features of the disease. In an attempt to do so, the Juvenile Diabetes Research Foundation (JDRF), the Endocrine Society, and the American Diabetes Association recently suggested the adoption of a new “Staging Classification System” for T1D that also integrates clinical aspects of disease progression. The disease is divided into three phases: stage 1: autoimmunity and/or normoglycemia or presymptomatic T1D; stage 2: autoimmunity and/or dysglycemia or presymptomatic T1D; and stage 3: autoimmunity and/or dysglycemia or symptomatic T1D. In these stages, there is a variable progression from beta cell autoimmunity to glucose intolerance and to the symptomatic stage ^30^. Whichever model is chosen to stratify the different phases of the disease, the means of detecting beta cell destruction and survival should be further investigated in order to evaluate their potential as biomarkers and design better therapeutic strategies adapted to the patient’s needs and according to the number of beta cells present in the pancreas at diagnosis.

## Remaining beta cell mass at onset and after diagnosis: beta cell destruction versus preservation

In the model described by Eisenbarth, only around 10% of beta cell mass remained at diagnosis ^19^. As many have discussed before, “diagnosis” might have different meanings depending on the time of disease progression. By definition, diagnosis is the “identification of the nature of an illness or other problem by examination of the symptoms”. Therefore, the keys are the stage at which disease progression symptoms appear and whether the patient is sufficiently aware of them. Consequently, every patient might be diagnosed at a different time point in the disease course ^31^. In addition, many patients still produce small amounts of C-peptide, especially if they are on their first 2 years post-diagnosis ^31^. An important study by Oram *et al.* showed that 80% of people with T1D for at least 5 years had detectable endogenous C-peptide, supporting the notion that after diagnosis an unknown number of beta cells remain in the pancreas ^32^. In accordance, no changes to the 2-hour Oral Glucose Tolerance Test (OGTT) were detected until 0.8 years prior to diagnosis in the Diabetes Prevention Trial-Type 1 (DPT-1), followed by a rapid decline in the OGTT response post-diagnosis ^26^. A decrease in stimulated C-peptide was also observed 6 months prior to the appearance of the first symptoms, with levels rapidly declining 3 months before the symptomatic phase ^24^. Moreover, C-peptide decline does not seem to occur at the same rate in adults versus children, the latter showing a 50% decline in the first year, whereas in the former the 1-year decline is set at 20% ^33^. Overall, different disease progression to diagnosis coupled with the heterogeneity of the disease makes it difficult to establish a standard for the number of remaining beta cells at diagnosis.

**Figure 1.**
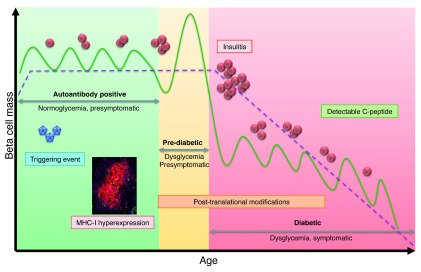
What does the type 1 diabetes scenario look like nowadays? Genetically predisposed individuals are exposed to a triggering event, which induces inflammation and upregulation of major histocompatibility complex class I (MHC-I) molecules in endocrine cells. Autoantibodies against islet antigens appear in the circulation during the autoantibody-positive phase. In this period, blood glucose levels are normal and there are no evident symptoms of the disease. During this chimera of normality, autoreactive T cells proliferate and start to infiltrate the islets, causing waves of beta cell death. In this period, an imbalance between beta cell death and survival generates small fluctuations in beta cell mass. The immune infiltration becomes apparent during the pre-diabetic phase, in which infiltrating cells actively destroy beta cells at a faster pace, a phenomenon that cannot be overcome by endogenous regulatory elements. Dysregulation of glucose levels starts to appear because of the inability of the remaining beta cells to produce enough insulin. In addition, post-translational modifications (PTMs) might arise as a consequence of endoplasmic reticulum stress and inflammation, potentiating the activation of cytotoxic CD8 ^+^ T cells and further beta cell destruction. PTMs might expand into the diabetic period, as inflammation is still present. At diagnosis, the number of cells infiltrating the islets is large and insulitis becomes more apparent. The decline in beta cell mass makes it impossible to control glucose levels, and the first symptoms of disease start to appear. During the first years of the diabetic period, C-peptide might still be detectable as some beta cells manage to survive the immune attack. However, as beta cell mass diminishes, so do inflammation and infiltration. Overall, fluctuations in beta cell mass over the years create a continuous relapsing-remitting phenotype with a more abrupt fall around the time of diagnosis, when a critical beta cell mass is reached.

The access to human pancreatic tissue samples has been critical to estimate the number of residual beta cells in T1D donors ^34, 35^. Studies emanating from the JDRF Network for Pancreatic Organ donors with Diabetes (nPOD) have shown that beta cell mass is very heterogeneous, even in non-diabetic individuals ^34^. In addition, recent reports have shown that a small number of patients with T1D still have remaining insulin-producing cells many years after diagnosis ^36, 37^, and this probably explains the presence of detectable C-peptide in some of these patients. Three interesting scenarios can be contemplated in light of recent findings: (1) several waves of beta cell destruction in a relapsing-remitting fashion over the pre-diabetic period and normal C-peptide production, (2) an abrupt fall in beta cell mass at diagnosis and abnormally low C-peptide levels, and (3) possible formation of new beta cells or proliferation during the diabetic phase (enough to produce a small amount of insulin and allow C-peptide detection) ( Figure 1).

As pointed out by many, there is a need for more insights into the pathology of human T1D in order to understand the sequence of events that might take place in the pancreas before and after diagnosis: how and when are beta cells destroyed? How and when do immune cells attack the islets? The answers to these questions will have enormous repercussions for clinical intervention before and after diagnosis.

## Hallmarks of the disease: insulitis, antigen specificity, MHC-I, and heterogeneity

The diabetes research community has relied heavily on animal models and post-mortem-donated human pancreatic tissue for their studies. Thus, most of what we know about the initial stages of autoimmune diabetes will be either snapshots from human pathology samples or data extrapolated from animal models. However, it is clear that the disease presents with autoantibodies before symptoms arise and that there is substantial islet inflammation (insulitis) ^38^, where an autoreactive destruction of insulin-producing cells by CD8 ^+^ T cells is ongoing ^37^.

The postulated presence of autoreactive CD8 ^+^ T cells in the islets of patients with T1D was proven by tetramer stainings of pancreatic sections ^37^. What was evident in this study was that the number of CD8 ^+^ T cells at the islets could not be completely explained by their reactivity toward islet antigens. The majority of CD8 ^+^ T cells in the insulitic lesion are not islet specific, and the proportion of specific cells could be as low as 1% ^39^. These observations are likely the result of two factors: (1) all autoreactive antigens have not been mapped yet, and this is due in part to PTM (discussed below), and (2) the ongoing inflammation in the islets likely recruits non-specific bystanders by factors such as chemokines expressed by lymphocytes and myeloid cells present at this site ^40, 41^. The importance of the specificity versus non-specificity of the T cells at this site is not clear at the moment, but the general hypothesis is that additive damage to the insulin-producing cells occurs via the non-specific release of beta cell-toxic cytokines.

As mentioned above, the majority of CD8 ^+^ T cells present in the insulitic lesion are not reactive against “known” beta cell antigens. T cells recognizing autoantigens in the thymus and bone marrow are deleted to avoid recognition of self-peptides in tissues and, consequently, autoimmunity. However, central tolerance does not account for potential PTMs that might arise in life. PTMs alter the primary sequence of the protein, which has a potential impact on MHC-peptide affinity or MHC–peptide–T cell receptor interactions ^42, 43^. PTMs can be mediated by enzymes like peptidyl arginine deiminase (PAD) or tissue transglutaminase 2 (TG2) or occur spontaneously ^44^; they can cause modifications of protein structure or their biological function or cause degradation. The activity of modifying enzymes can be enhanced by endoplasmic reticulum (ER) stress or other environmental triggers, among them viral or bacterial infections. Alternatively, beta cell antigens could also trigger the first autoimmune attack, in turn activating modifying enzymes and ultimately propagating autoreactivity, epitope spreading, and the perpetuation of the autoimmune attack. As part of the immune response, modified antigens could be processed and presented by antigen-presenting cells to T cells and B cells.

The first evidence for PTM occurring in T1D was the observation of T cell reactivity toward an altered insulin epitope where a non-native disulfide bond had formed through spontaneous oxidation ^45^. Since then, both PAD-meditated citrullination ^46^ and TG2-mediated transglutamination ^47^ of islet antigens have been found in patients with T1D. For example, one of the major autoantigens, GAD65, can be found in both transglutaminated and citrullinated forms, thus being modified by both major PTM enzymes ^48^. The high metabolic rate in beta cells makes them highly susceptible to ER stress ^49, 50^. This, combined with the islet inflammation in T1D, leads to a high probability of PTMs occurring. Thus, PTMs of islet antigens could be a major factor in the non-linearity of T1D progression (discussed above), as the self-propagation of the inflammation and cellular stress could lead to the genesis of neo-antigens that ultimately would increase the rate of autoimmune beta cell destruction.

Another histopathologic hallmark of T1D besides the lymphocyte infiltrate in the islets is the hyperexpression of MHC-I on endocrine cells. Since the first observation in the 1980s, islets from T1D subjects have been found to overexpress MHC-I and interferon-α in several studies ^51, 52^. However, the only evidence for MHC-I overexpression is from immunostaining of tissue sections ^18, 37, 53^. A recent article tried to find quantitative evidence for an increase in MHC-I at the mRNA and protein levels ^54^. In this study, MHC-I hyperexpression was detected by immunohistochemical methods, but the limited comparisons performed failed to correlate with quantitative mRNA detection methods, questioning the relevance of previous observations. However, it is known that mRNA is rapidly degraded under the harsh conditions present in the pancreas. In addition, several laboratories have reported the presence of MHC-I hyperexpression in T1D donors by using different antibodies in different specimens preserved under different conditions (frozen versus formalin-fixed paraffin-embedded) ^18, 37, 53, 55, 56^. This overexpression of MHC molecules and type I interferons is enigmatic but throws suspicion on a possible viral infection of the pancreas as a contributing factor to T1D. Common to both insulitis and MHC-I expression is heterogeneity in the distribution throughout the organ. The histopathologic features of the pancreas are lobular, and so large parts can be affected while some lobules remain undisturbed ^56, 57^. This asymmetrical anatomical distribution of the lesions could be a consequence of a viral infection or a feature of an anatomical distribution network such as the nervous ^58^, vascular ^59– 61^, or acinar-ductal ^62^ systems.

The key to understanding the initial autoimmune events in T1D will be to know why beta cells upregulate MHC molecules and what controls the initial lymphocytic infiltration of the islets. Is MHC-I hyperexpression just a pseudophenomenon in the wake of insulitis, or does it have a disease-propagating value? Is antigen a main gatekeeper for CD8 ^+^ T cells in the developing insulitic lesion, or are other, less appreciated signals (e.g. innate, neuronal, or vascular) at play?

## Conclusions

Environmental triggers continue to be enigmatic, but longitudinal studies that are following patients over many years could shed light on potential environmental influence if a sufficiently strong correlation with disease progression is found. The ambitious pancreas tissue biobank projects and novel biopsy studies have already, shortly after their inception, changed our view of the disease. They have allowed investigators around the world to look into the pancreas and have a glimpse of the residual beta cell mass in the pre-diabetic phase, at diagnosis, and many years thereafter. The evolution of the field during the last few decades has proven that our views of the disease’s cause and progression of inflammation are in constant need of reassessment. Further efforts should be made in order to identify potential new autoantigens and PTMs and characterize bystander inflammation in order to know what facilitates immune cell entry into the islets. In this short review, we aimed to highlight some of the major remaining questions in T1D research, areas that we believe may be key to solving this enigmatic disease.

## Abbreviations

ER, endoplasmic reticulum; JDRF, Juvenile Diabetes Research Foundation; MHC, major histocompatibility complex; OGTT, Oral Glucose Tolerance Test; PAD, peptidyl arginine deiminase; PTM, post-translational modification; T1D, type 1 diabetes; TG2, transglutaminase 2.
